# 200 Sentinel lymph node biopsies without axillary lymph node dissection – no axillary recurrences after a 3-year follow-up

**DOI:** 10.1038/sj.bjc.6601765

**Published:** 2004-04-06

**Authors:** R Reitsamer, F Peintinger, E Prokop, L Rettenbacher, C Menzel

**Affiliations:** 1 Department of Senology, University Hospital Salzburg, Paracelsus Private Medical School Salzburg, Muellner Hauptstrasse 48, 5020 Salzburg, Austria; 2 Department of Gynecology and Obstetrics, General Hospital Leoben, Vordernbergerstrasse 42, 8700 Leoben, Austria; 3 Department of Pathology, University Hospital Salzburg, Paracelsus Private Medical School Salzburg, Muellner Hauptstrasse 48, 5020 Salzburg, Austria; 4 Department of Nuclear Medicine, University Hospital Salzburg, Paracelsus Private Medical School Salzburg, Muellner Hauptstrasse 48, 5020 Salzburg, Austria

**Keywords:** Sentinel lymph node, Sentinel lymph node biopsy, breast cancer, axillary lymph node dissection

## Abstract

The aim of this study is to evaluate the rate of axillary recurrences in sentinel lymph node (SLN)-negative breast cancer patients after sentinel lymph node biopsy (SLNB) alone without further axillary lymph node dissection (ALND). Between May 1999 and February 2002, 333 consecutive patients with primary invasive breast cancer up to 4 cm and clinically negative axillae were entered into this prospective study. Sentinel lymph nodes were identified using the combined method with blue dye (Patent blue V®) and technetium 99m-labelled albumin (Nanocoll®). Sentinel lymph nodes were examined by frozen sections, standard haematoxylin and eosin staining and immunohistochemistry staining. In SLN-positive patients, ALND was performed. Sentinel lymph node-negative patients had no further ALND. The SLN identification rate was 98.5% (328 out of 333). In all, 128 out of 328 (39.0%) patients had positive SLNs and complete ALND. A total of 200 out of 328 (61.0%) patients were SLN negative and had no further ALND. The mean tumour size of SLN-negative patients was 16.5 mm. The mean number of SLNs removed was 2.1 per patient. There were no local or axillary recurrences at a median follow-up of 36 months. The absence of axillary recurrences after SLNB without ALND in SLN-negative breast cancer patients supports the hypothesis that SLNB is accurate and safe while providing less surgical morbidity than ALND. Short-term results are very promising that SLNB without ALND in SLN-negative patients is an excellent procedure for axillary staging in a cohort of breast cancer patients with small tumours.

Axillary lymph node dissection (ALND) has been the surgical standard treatment of the axilla for breast cancer patients for decades and is about to be replaced by sentinel lymph node (SLN) biopsy (SLNB) for patients with early-stage breast cancer. The rationales for ALND are the exact staging and prognosis, the regional control in the axilla and the possibility of survival improvement (Fisher *et al*, 1980; Harris and Osteen, 1985; Petrek and Blackwood, 1995). The extent of the axillary lymph node involvement is one of the most important independent prognostic factors for recurrence and survival in patients with invasive breast cancer (Carter *et al*, 1989; Rosen *et al*, 1989, 1993; Rosen and Groshen, 1990; NIH Consensus Conference, 1991; Mustafa *et al*, 1998). However, ALND is associated with major problems such as pain, restriction of arm motion or chronic lymphedema. One of the most important advances in the surgical treatment of breast cancer is the introduction of SLNB. Sentinel lymph node biopsy is an innovative method for axillary staging in breast cancer patients. Many studies have shown that SLNB can accurately predict axillary lymph node status. Sentinel lymph node biopsy is a minimally invasive surgical technique for axillary staging and has the potential to reduce the morbidity of the surgical procedure (Petrek *et al*, 2001; Temple *et al*, 2002; Peintinger *et al*, 2003; Schijven *et al*, 2003). The overall risk of axillary lymph node metastases in invasive breast cancer is decreasing with the early detection of small tumours. The increasing frequency of routine mammograms and the awareness of women leads to the early detection of small breast carcinomas. The probability of axillary lymph node involvement in those patients is 30–40% (Lin *et al*, 1993; Petrek and Blackwood, 1995; Cady *et al*, 1996). These patients possibly could benefit from ALND. The remaining 60–70% with negative axillary lymph nodes may thus have an unnecessary ALND and suffer from minor to major short- and long-term morbidity of ALND. Many studies have shown that SLNB can identify axillary lymph node involvement in most patients (Krag *et al*, 1993, 1998; Giuliano *et al*, 1994, 1997; O'Hea *et al*, 1998; Nieweg *et al*, 2001). The aim of this study was to evaluate the rate of axillary recurrences in SLN-negative patients after SLNB alone, without further ALND.

## PATIENTS AND METHODS

A total of 333 consecutive patients with invasive breast carcinomas was included in this prospective study between May 1999 and February 2002. Diagnosis of invasive breast carcinoma was performed by core needle biopsy prior to surgery in all cases. All patients had clinically negative axillae. Patients, who had *in situ* carcinomas, multicentric carcinomas or patients with locally advanced disease or tumours larger than 4 cm or clinically positive axillary lymph nodes were excluded from the study. Informed consent was obtained from all patients. Patient characteristics are summarised in Table 1

**Table 1:** Patients characteristics

	**SLNB only (*n*=200)**	**%**	**SLNB+ALND (*n*=128)**	**%**
*Number of nodes removed*				
Min	1		7	
Max	8		45	
Median	2		19	
				
*Histology*				
Invasive ductal carcinoma	178	89.0%	116	90.6%
Invasive lobular carcinoma	22	11.0%	12	9.4%
				
*Tumour size (mm)*				
Min	1		5	
Max	50		55	
Range	49		50	
Mean	16.5		20.45	
Median	15		19	
				
*T stage*				
T1a	7	3.5%	1	0.8%
T1b	24	12.0%	3	2.3%
T1c	118	59.0%	66	51.6%
T2	51	25.5%	57	44.5%
T3	0		1	0.8%
				
*Grading*				
G1	26	13.0%	4	3.1%
G2	124	62.0%	91	71.1%
G3	50	25.0%	33	25.8%
				
*Hormone receptor status*				
ER neg/PR neg	27	13.5%	10	7.8%
ER neg/PR pos	2	1.0%	0	
ER pos/PR pos	157	78.5%	101	78.9%
ER pos/PR neg	14	7.0%	17	13.3%
				
*Menopausal status*				
Premenopausal	41	20.5%	40	31.3%
Postmenopausal	159	79.5%	88	68.7%

. For the identification of SLNs, the combined technique using blue dye and radioactive tracer was performed. Technetium-99m-labelled albumin (Nanocoll®, Sorin Biomedica, Saluggia, Italy) was injected peritumorally 16–18 h before surgery at a dose of 30–60 MBq by the nuclear medicine physician if the tumour was palpable. The injection was performed by ultrasound guidance if the tumour was not palpable. A dynamic lymphoscintigraphy was performed after injection and before surgery. The skin overlying the hot spot was marked with a skin marker. Subareolar subcutaneous injection of 2 ml of blue dye (Patent Blue V®, Laboratoire Guerbet, Aulnay-sous-Bois, France) was performed in the operating room after draping of the patient, and exactly 5 min after injection of the blue dye the axillary incision near the hot spot was performed. Careful and bloodless dissection was performed to identify the blue lymphatic channels leading to the blue SLN. Additionally, a gamma probe (C-Trak, Care Wise, Morgan Hill, CA, USA) was used to identify the hot SLN. All hot and blue nodes were excised and frozen sections were made. Subsequently, breast surgery was performed as indicated. Axillary lymph node dissection was done in the same surgical procedure if SLNs could not be identified, or if SLNs were positive for metastases in frozen sections. No further ALND was performed, if SLNs were negative in frozen sections. If negative SLNs converted positive in permanent haematoxylin and eosin (H&E)-stained sections or immunohistochemistry (IHC)-stained sections, a secondary ALND had to be performed. Sentinel lymph node biopsy alone, without ALND, was performed exclusively if SLNs were negative in frozen sections and in H&E-stained slides and in IHC-stained slides. The histopathologic examination of SLNs was performed according to the Austrian Pathology Society's consensus conference statement. SLNs were cut in 2–3 mm slices, from which 2–3 frozen sections were obtained. Slices were embedded in paraffin and serially cut in 250 *μ*m levels. From each level, one H&E-stained slide and one IHC-stained slide using cytokeratin cocktail (AE1/AE3) were examined if the H&E-stained slide was negative for metastases. Adjuvant treatment of SLN-negative patients consisted of tamoxifen in most oestrogen receptor-positive postmenopausal women, and consisted of LHRH analogues combined with tamoxifen or anastrozole in premenopausal oestrogen receptor-positive patients. Oestrogen receptor-negative patients received adjuvant chemotherapy. All patients with breast-conserving surgery received radiotherapy to the whole breast after surgery, but no radiotherapy was given to the axilla. Postsurgical follow-up consisted of clinical controls every 3 months in combination with sonography of the breast and the axilla. Mammograms, X-ray and abdominal sonography were performed annually.

## RESULTS

A total of 333 patients were included in the study. The SLN could be identified in 328 out of 333 patients, calculating an identification rate of 98.5%. SLNs were positive for metastases in 128 out of 328 patients (39.0%). In all, 104 out of 128 patients (81.3%) had positive SLNs in frozen sections and underwent ALND immediately after SLNB in the same surgical procedure. In nine out of 128 patients (7.0%) SLNs were negative in frozen sections, but positive in permanent H&E staining, and in further 15 out of 128 patients (11.7%) SLNs converted positive in IHC staining. Hence, in 24 patients a secondary ALND had to be performed after receipt of the final pathological report. That means, 18.7% (24 out of 128) of all patients with positive SLNs, respectively 10.7% (24 out of 224) of all patients with primarily negative SLNs, respectively 7.3% (24 out of 328) of all patients, who had a successful SLNB, had to undergo secondary ALND. In total, 224 out of 328 patients (68.3%) were SLN negative in frozen sections, but dropped to 215 out of 328 (65.5%) after H&E staining in permanent sections, and totalled in 200 out of 328 (61.0%) SLN-negative patients after addition of IHC staining (Table 2

**Table 2:** Sentinel lymph node histopathology

	***n***	**%**
Total number of patients	333	100.0%
SLN identification	328/333	98.5%
SLN pos (FS)	104/328	
SLN pos (FS and H&E)	113/328	
SLN pos (FS, H&E and IHC)	128/328	39.0%
SLN neg (FS)	224/328	
SLN neg (FS and H&E)	215/328	
SLN neg (FS, H&E and IHC)	200/328	61.0%

FS=frozen section, H&E=haematoxylin and eosin staining, IHC=immunohistochemistry staining.

). Consecutively, 15 out of 215 patients (7.0%) converted from negative to positive and were upstaged by the addition of IHC staining. Exclusively, the 200 patients whose SLNs were negative in frozen sections and permanent H&E-stained sections and in IHC staining had SLNB alone without further ALND. The mean number of SLNs removed was 2.1 per patient and the mean number of lymph nodes removed in ALND was 20.8 per patient. The SLN was the only positive node in 77 out of 128 patients (60.2%). The mean tumour size was 20.45 mm in SLN-positive patients and 16.5 mm in SLN-negative patients. The median follow-up period was 36 months with a maximum follow-up time of 56 months and a minimum follow-up time of 22 months. No local or axillary recurrence could be observed in the 200 patients, who underwent SLNB without ALND.

## DISCUSSION

The present standard of care for treatment of early-stage invasive breast cancer is partial or total mastectomy and ALND of levels I and II, and occasionally of level III. About 30–40% of patients have positive axillary lymph nodes. The remaining 60–70% of patients are lymph node negative and may therefore be overtreated with ALND, with the disadvantage of early and late complications as seroma, pain, limited arm motion, numbness or lymph oedema of the arm and breast (Kuehn *et al*, 2000). Sentinel lymph node biopsy is a minimally invasive surgical procedure with significant lower morbidity than ALND (Peintinger *et al*, 2003; Schijven *et al*, 2003). The accuracy of SLNB for axillary staging has been confirmed in many studies (Krag *et al*, 1993; Giuliano *et al*, 1994, 1997; Krag *et al*, 1998; O'Hea *et al*, 1998; Nieweg *et al*, 2001). The long-term outcome of SLNB without ALND has not yet been evaluated and prospective randomised trials comparing SLNB alone *vs* SLNB plus ALND in SLN-negative patients as the American NSABP-B 32 trial or the European ALMANAC trial are in the recruitment phase. Few data exist on the local control of SLNB and there are only a few reports on SLNB alone without further ALND to date (Giuliano *et al*, 2000; Roumen *et al*, 2001; Schrenk *et al*, 2001; Chung *et al*, 2002). Axillary recurrences, as reported in those studies, range between 0 and 1.4% and follow-up periods range between 22 and 39 months. A recent study (Chung *et al*, 2002) reported on 208 patients with SLNB alone with a median follow-up of 26 months. In this study, three patients developed axillary recurrences after a negative SLNB, estimating a false-negative rate of 1.4%. In this study, 60% of patients received adjuvant systemic therapy. As nearly all of our patients received adjuvant sytemic treatment and all patients with breast-conserving surgery received radiotherapy to the whole breast, but not to the axilla, it cannot be stated as to what extent adjuvant treatment and radiotherapy contributed to the negative axillary failure rate in our group of patients. In our study, we have a median follow-up period of 36 months and no axillary recurrence could be observed. This may be a rather short follow-up period, but in a study on outcome of axillary recurrences after ALND (Newman *et al*, 2000) a median time interval of 19 months for local recurrence after ALND is reported. Axillary recurrence after ALND ranges between 0 and 3% (Recht and Houlihan, 1995). We could observe no axillary recurrence in 200 patients with a median follow-up of 36 months after SLNB only. If we had missed the true SLN and if we had an unknown false-negative rate, we should have observed 2–12% of patients (Kjaergaard *et al*, 1985; Senofsky *et al*, 1991) with axillary recurrences, which were 4–24 patients. All of our patients were SLN negative in frozen sections, H&E and IHC staining. As the impact of micrometastases identified by IHC is still controversial, we carried out ALND in all patients with IHC-positive SLNs according to our protocol. IHC was positive for micrometastases in 15 out of the 328 patients, but we could not find any further metastases in non-SLNs. A study from the Lee Moffitt Cancer Center (Jakub *et al*, 2002) suggested that ALND should be performed in patients with SLNs positive by CK-IHC only to reduce the false-negative rate. To date, there is no definite answer as to how to treat patients with micrometastases in SLNs. ACOSOG Z0010 will answer the question of micrometastases in SLNs and bone marrow in the near future.

The results of our study confirm the accuracy of SLNB and SLN-negative patients with SLNB alone are not at risk for axillary recurrences in a short-term follow-up. However, as long as prospective randomised studies are not available, ALND should not be abandoned as the standard of care for SLN-negative patients.
